# Supplementary material for: Strain-driven Kovacs-like memory effect in glasses
Source: Nat Commun. 2023 Dec 18;14:8407. doi: 10.1038/s41467-023-44187-x (PMC10728148; doi:10.1038/s41467-023-44187-x)
Supplement: Supplementary file 1 — Supplementary Information [file 41467_2023_44187_MOESM1_ESM.pdf]

Supplementary Information for

Strain-driven Kovacs-like memory effect in glasses

Yu Tong[1], Lijian Song[1,2,*], Yurong Gao[1], Longlong Fan[3], Fucheng Li[4], Yiming Yang[3], Guang Mo[3], Yanhui Liu[4], Xiaoxue Shui[5], Yan Zhang[1], Meng Gao[1], Juntao Huo[1,2], Jichao Qiao[6], Eloi Pineda[7,*], Jun-Qiang Wang[1,2,*]

[1] *CAS Key Laboratory of Magnetic Materials and Devices, and Zhejiang Province Key Laboratory of Magnetic Materials and Application Technology, Ningbo Institute of Materials Technology and Engineering, Chinese Academy of Sciences, Ningbo, 315201, China*

[2] *Center of Materials Science and Optoelectronics Engineering, University of Chinese Academy of Sciences, Beijing 100049, China*

[3] *Beijing Synchrotron Radiation Facility, Institute of High Energy Physics, Chinese Academy of Sciences, Beijing 100049, People's Republic of China*

[4] *Institute of Physics, Chinese Academy of Sciences, Beijing 100190, China*

[5] *Ningbo Institute of Materials Technology and Engineering, Chinese Academy of Sciences, Ningbo 315201, China*

[6] *School of Mechanics, Civil Engineering and Architecture, Northwestern Polytechnical University, Xi'an, 710072, PR China*

[7] *Department of Physics, Institute of Energy Technologies, Universitat Politècnica de Catalunya, Barcelona, 08019, Spain*

* Correspondence: jqwang@nimte.ac.cn, eloi.pineda@upc.edu, songlj@nimte.ac.cn

Contents

1. Sample fabrication and dynamic properties......2

1.1. Sample Fabrication......2

1.2. Dynamic Characterization......2

2. Peak time t_p......3

3. Relaxation decoupling......4

4. Activation energy E_a......5

5. Reversible deformation......8

6. *In-situ* synchrotron X-ray diffraction characterization......9

7. Anelastic recovery......12

1. Sample fabrication and dynamic properties

1.1. Sample fabrication

The master alloys of $Ti_{16.7}Zr_{16.7}Hf_{16.7}Cu_{16.7}Ni_{16.7}Be_{16.7}$, $(Fe_{11}Zr_1)_{91.2}B_{8.8}$ (at%) were prepared by arc melting. The metallic glass ribbons were fabricated by the melt spinning with a tangent velocity of approximately 40 m/s. Polyvinyl chloride films were purchased from the manufacturer.

1.2. Dynamic characterization

The dynamical mechanical behavior of the three glasses was measured using the dynamic thermomechanical analysis apparatus (TA-DMA Q800).

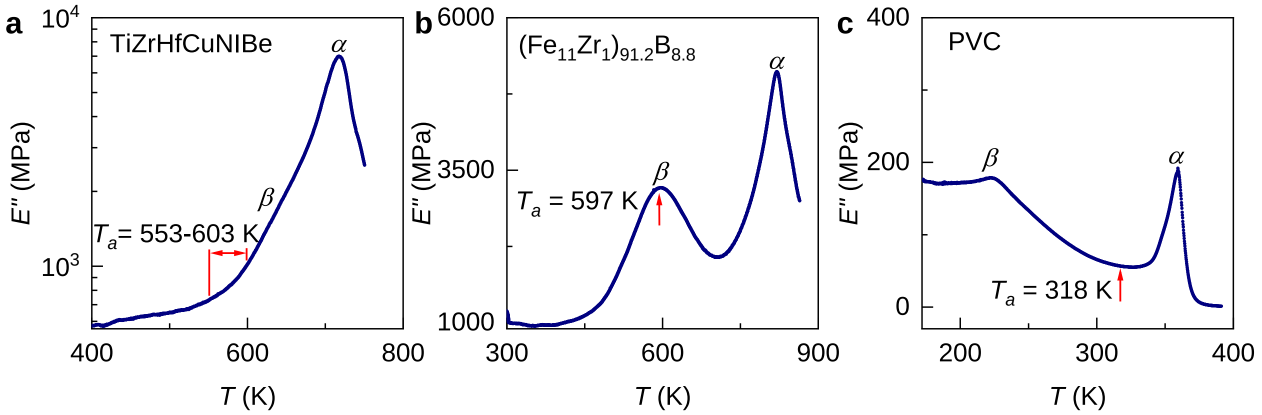

Supplementary Figure 1: Dynamic characterization of three glasses. Temperature dependence of loss modulus for **a** TiZrHfCuNiBe MG, **b** $(Fe_{11}Zr_1)_{91.2}B_{8.8}$MG, and **c** PVC polymer. The heating rate is 5 K/min, and the driving frequency is 1 Hz. β, α represent the beta and main relaxation, respectively. The tested temperatures T_a for three glasses are marked with red arrows. Source data are provided as a Source Data file.

2. Peak time t_p

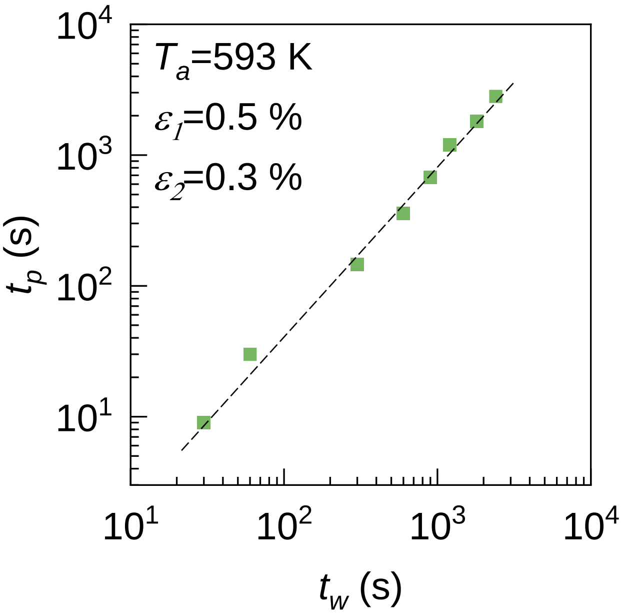

Supplementary Figure 2: The linear relationship between the peak time t_p and preloading time t_w in TiZrHfCuNiBe MG at 593 K. Source data are provided as a Source Data file.

3. Relaxation superposition

Supplementary Fig. 3b shows the effect of the conditions of the pre-relaxation tests on the relaxation times, and it can be found that the relaxation times can be classified into two different regions separated by several orders of magnitude. It implies that the relaxation can be decoupled into two modes: **mode I** with the longer relaxation time τ_1 produces the stress decay, while **mode II** with the shorter relaxation time τ_2 is responsible for the stress increase, as depicted schematically in Supplementary Fig. 3a.

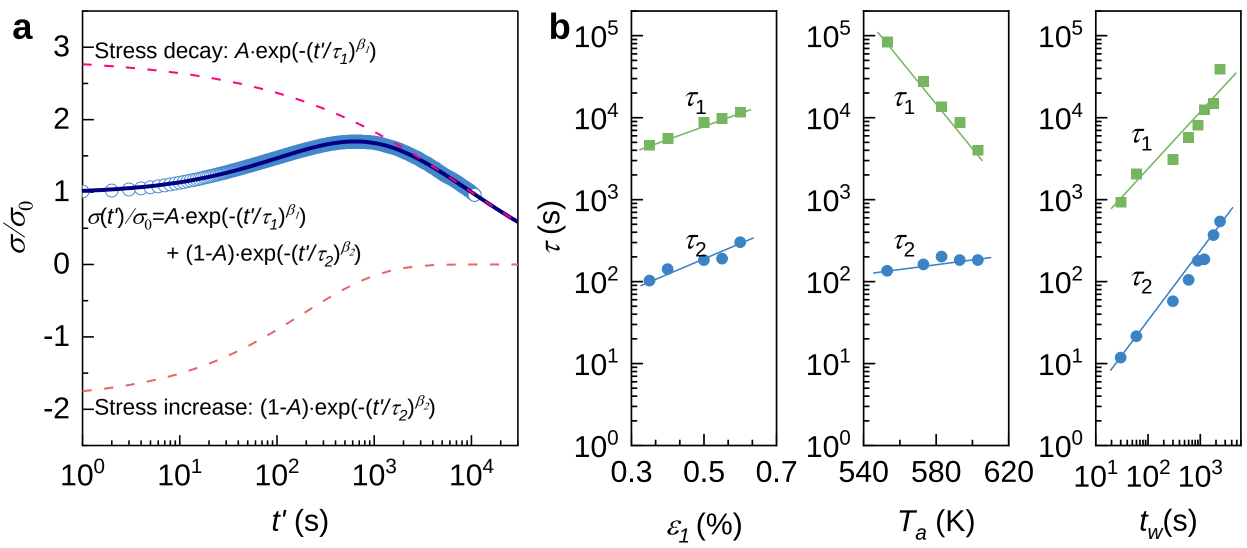

Supplementary Figure 3: Relaxation superposition. a The schematic profile of relaxation decoupling in Kovacs-like memory effect. The experimental data (hollow blue circles) can be characterized by double KWW expression (solid blue line). **b** The fitted characteristic relaxation times (τ_1, τ_2) as the function of preloading strain ε_1, test temperature T_a and preloading time t_w. Source data are provided as a Source Data file.

4. Activation energy E_a

The single-step stress relaxation can be described as a function of time by the standard Kohlrausch-Williams-Watts (KWW) model: $\frac{\sigma(t)}{\sigma_0} = \exp\left(-\left(\frac{t'}{\tau_{kww}}\right)^{\beta_{kww}}\right)$. Here, $\sigma(t)$ and σ_0 respectively represent the immediate and initial stress during the last step, t' (=t-t_w) is the time elapsed since the start of the final relaxation test at t_w.

To capture instant information during single stress relaxation, we take a twice logarithm on both sides of the KWW equation to obtain the following equation:

$$\ln\left(\ln\left(\frac{\sigma(0)}{\sigma(t)}\right)\right) = \beta_{kww} \cdot \ln(t) - \beta_{kww} \cdot \ln(\tau_{kww})$$

Then the value of instant β_{kww} and instant τ_{kww} can be estimated by taking the derivative of $\ln\left(\ln\left(\frac{\sigma(0)}{\sigma(t)}\right)\right)$ to $\ln(t)$, as shown in Supplementary Figs. 4a, b. During the initial relaxation stage, β_{kww} is close to 0.5, a phenomenon often associated with the diffusion of defects[1-4]. The instant activation energy Ea during the preloading state was estimated by the Arrhenius equation: $\tau_{kww} = \tau_E \exp\left(\frac{E_a}{RT}\right)$, where τ_E and R represent the pre-exponential factor and gas constant, respectively. The corresponding fitting curves of TiZrHfCuNiBe are shown in Supplementary Fig. 4c.

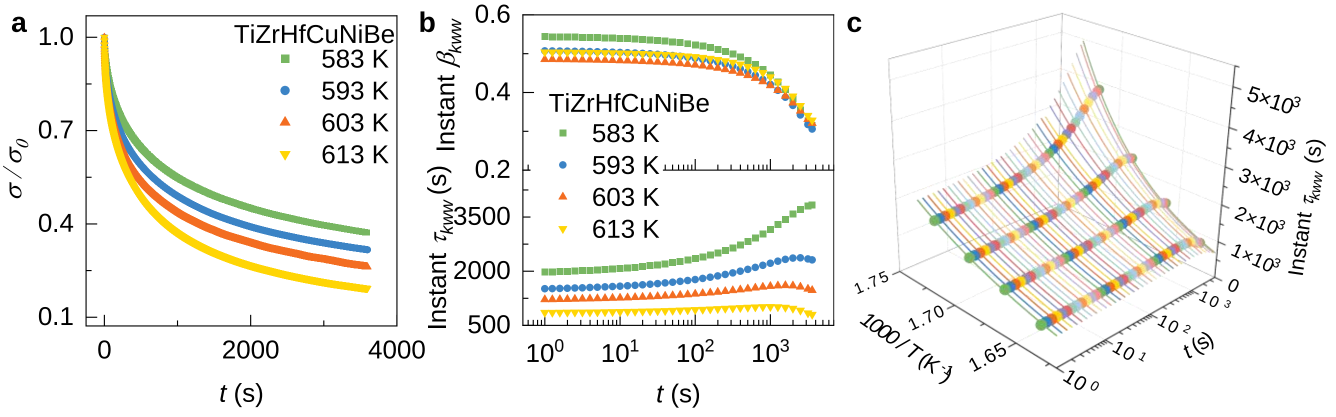

Supplementary Figure 4: Relaxation kinetics of TiZrHfCuNiBe MG. a Normalized stress as a function of time at various temperatures during the preloading step in the TiZrHfCuNiBe MG; **b** The evolution of instant relaxation parameters (top: instant β_{kww}, bottom: instant τ_{kww}) with preloading time at various temperatures for TiZrHfCuNiBe MG; **c** τ_{KWW} as a function of 1000/T (tested

temperature) for various preloading times t for TiZrHfCuNiBe MG. The solid lines are Arrhenius fitting curves. Source data are provided as a Source Data file.

The analogous calculation processes for $(Fe_{11}Zr_1)_{91.2}B_{8.8}$ and PVC glasses are presented in Supplementary Fig. 5 and Supplementary Fig. 6, respectively.

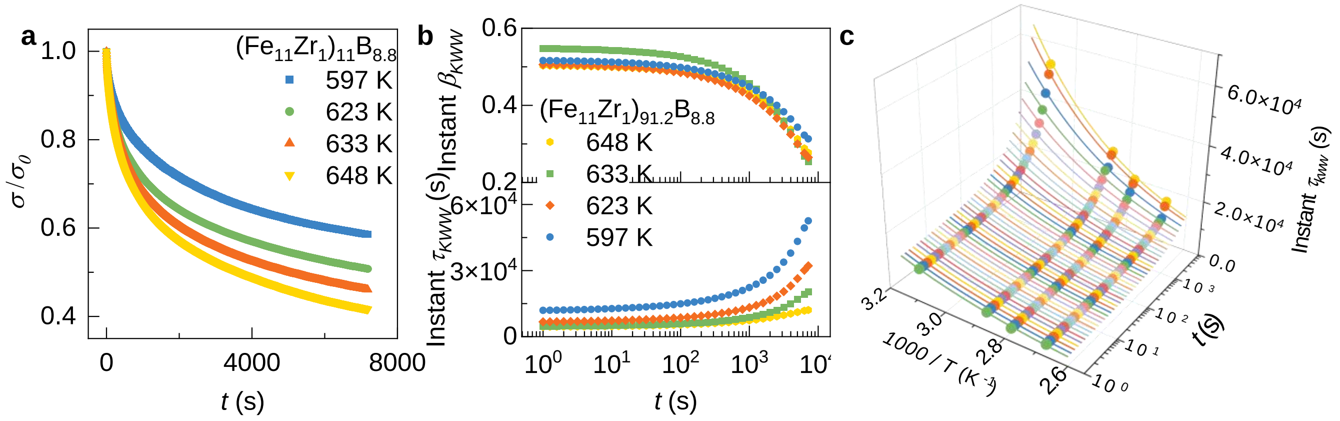

Supplementary Figure 5: Relaxation kinetics of $(Fe_{11}Zr_1)_{91.2}B_{8.8}$ MG. a Normalized stress as a function of time at various temperatures during the preloading step in the $(Fe_{11}Zr_1)_{91.2}B_{8.8}$ MG. **b** The evolution of instant relaxation parameters (top: instant β_{kww}, bottom: instant τ_{kww}) with preloading time at various temperatures for $(Fe_{11}Zr_1)_{91.2}B_{8.8}$ MG; **c** τ_{KWW} as a function of 1000/T for various preloading times t for $(Fe_{11}Zr_1)_{91.2}B_{8.8}$ MG. The solid lines are Arrhenius fitting curves. Source data are provided as a Source Data file.

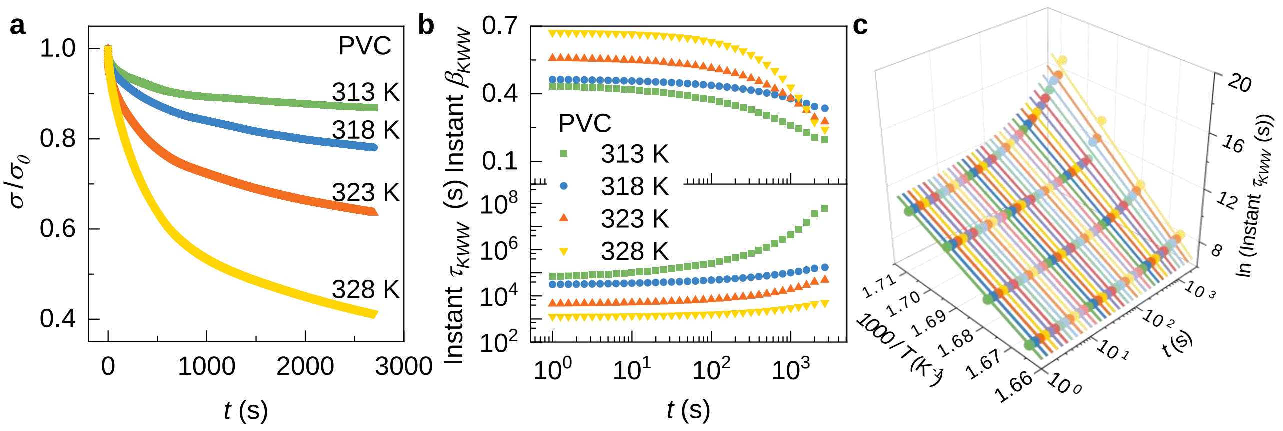

Supplementary Figure 6: Relaxation kinetics of PVC. a Normalized stress as a function of time at various temperatures during the preloading step in the PVC; **b** The evolution of instant relaxation parameters (top: instant β_{kww}, bottom: instant τ_{kww}) with preloading time at various temperatures for PVC; **c** τ_{KWW} as a function of 1000/*T* for various preloading times *t* for PVC. The solid lines are Arrhenius fitting curves. Source data are provided as a Source Data file.

Supplementary References

1 Brey, J. J. & Prados, A. Low-temperature relaxation in the one-dimensional Ising model. *Physical Review E* **53**, 458 (1996).

2 Shore, J. E. & Zwanzig, R. Dielectric relaxation and dynamic susceptibility of a one-dimensional model for perpendicular-dipole polymers. *The Journal of Chemical Physics* **63**, 5445-5458 (1975).

3 Brey, J. & Prados, A. Stretched exponential decay at intermediate times in the one-dimentional Ising model at low temperatures. *Physica A: Statistical Mechanics and its Applications* **197**, 569-582 (1993).

4 Spohn, H. Stretched exponential decay in a kinetic Ising model with dynamical constraint. *Communications in mathematical physics* **125**, 3-12 (1989).

5. Reversible deformation

Supplementary Fig. 7 displays the strain recovery curves upon stress removal (switching from $\sigma > 0$ to $\sigma = 0$), highlighting the presence of three strain components: pure elastic strain (ε_{el}), anelastic strain (ε_{an}), and viscoplastic strain (ε_{vp}). Anelastic deformation (ε_{an}) refers to the time-dependent and recoverable strain observed in a material after the removal of applied stress. In contrast, viscoplastic deformation (ε_{vp}) signifies permanent deformation that cannot be recovered upon stress removal.

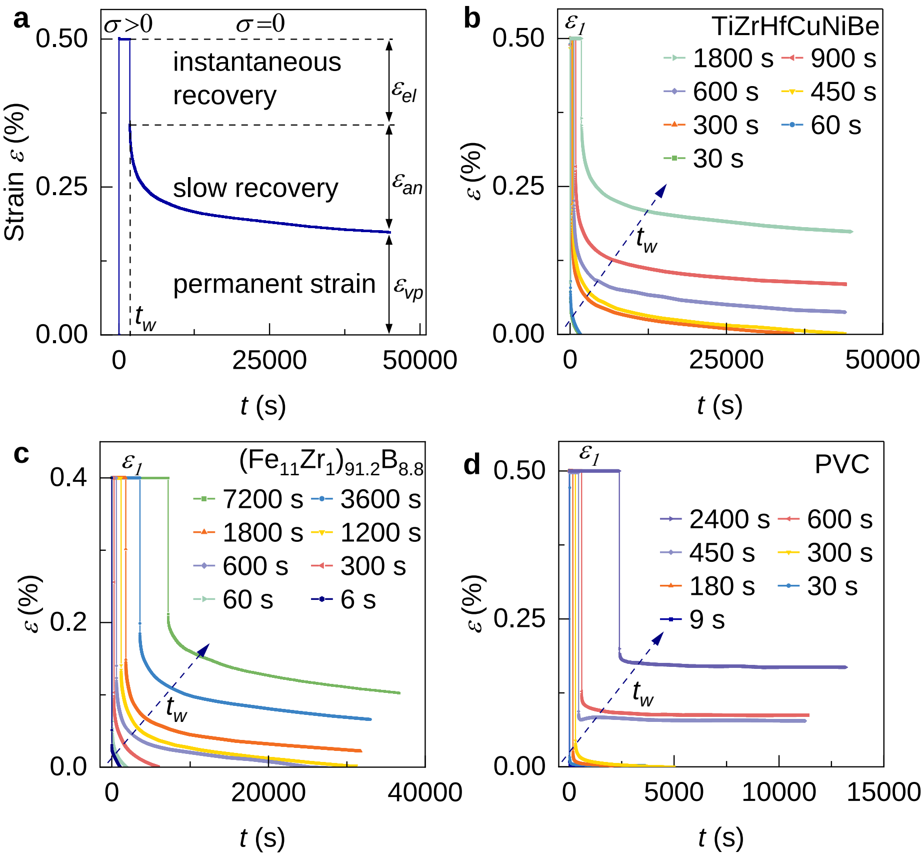

Supplementary Figure 7: Reversible deformation. **a** Schematic of the three deformation components observed in recovery experiments without applied force after stress relaxation below T_g (ε_{el}: elastic, ε_{an}: anelastic, ε_{vp}: viscoplastic). Fraction of strain recovery ($1 - \varepsilon_{vp} / \varepsilon_1$) of **b** TiZrHfCuNiBe MG at 573 K with a preloading strain ε_1 of 0.5%. **c** $(Fe_{11}Zr_1)_{91.2}B_{8.8}$ MG at 597 K with a preloading strain ε_1 of 0.4%. **d** PVC at 318 K with a preloading strain ε_1 of 0.5%. The test time of strain recovery for TiZrHfCuNiBe, $(Fe_{11}Zr_1)_{91.2}B_{8.8}$, and PVC are 43200 s, 30000 s and 11000s, respectively. Source data are provided as a Source Data file.

6. *In-situ* synchrotron X-ray diffraction characterization

The local structural characterization was performed in China's Beijing synchrotron radiation facility. The *in-situ* experiments were conducted in the synchrotron X-ray beam at 3W1, which utilized monochromatic synchrotron radiation with a wavelength of 0.2065 Å (energy of 60.037 keV). The diffraction experiments were carried out in the Debye-Scherrer geometry. The layout of the experimental setup is shown in Supplementary Fig. 8a. The incident beam had a well-collimated cross-section of 0.8×0.8 mm^2. Diffusion patterns were collected using an iRay Mercu 1717HS image plate detector, which had a resolution of 3072×3072 pixels, with each pixel corresponding to a size of 139×139 μm^2. The detector was carefully positioned orthogonal to the x-ray beam, and the distance between the 2D detector and the sample was adjusted to about 280 mm in terms of covering a high-Q range up to 16.5 $Å^{-1}$ (where $Q = 4\pi\sin\theta/\lambda$).$\theta$ is half the scattering angle, and λ is the incident wavelength. The samples were exposed to the incident beam for 0.5 seconds during the experiments. Each total diffusion pattern was fully integrated to achieve the diffraction data and the Fourier transformations from $I(Q)$ to $G(r)$ were conducted using the PDFgetx3 program.

The specimens of amorphous ribbon were subjected to tension using the Linkam TST350 straining system. Supplementary Fig. 8b shows the loading protocol for tensile stress relaxation with two different applied strains ($\varepsilon_1 = 0.5\%, \varepsilon_1 = 0.3\%$) at a temperature of 593 K. The tensile strain was increased in increments of 200%/min, and the gauge length of Linkam TST350 was 40 mm. The stress evolution corresponding to the two-step process is shown in Supplementary Fig. 8c.

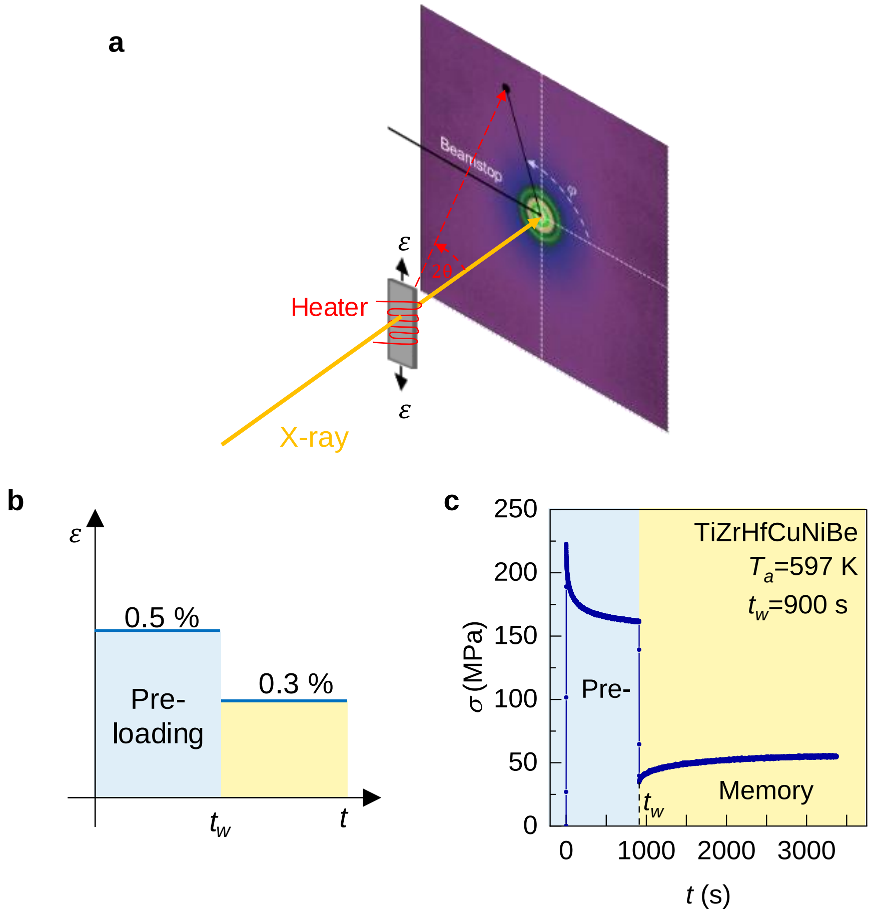

Supplementary Figure 8. *In-situ* synchrotron X-ray diffraction. a Schematic of the *in-situ* synchrotron X-ray scattering experiments. **b** The schematic of tensile stress relaxation with two different applied strains. **c** The flow stress for two-step relaxation ($\varepsilon_1 = 0.5$ %, $\varepsilon_2 = 0.3$ %) at 593 K with the preloading time t_w =900 s. The initial strain $\varepsilon_0 = 0$. Source data are provided as a Source Data file.

Supplementary Table 1. The possible nearest-neighbor pairs in $Ti_{16.7}Zr_{16.7}Hf_{16.7}Cu_{16.7}Ni_{16.7}Be_{16.7}$ metallic glass and their theoretical inter-atomic bond lengths R_{ij}^{0}(Å). Atomic radii (Ti-1.47 Å, Zr-1.60 Å, Hf-1.59 Å, Cu-1.28 Å, Ni-1.25 Å and Be-1.13 Å). w_{ij} denotes the weight factors of the atomic pairs calculated at $Q = 0Å^{-1}$. Source data are provided as a Source Data file.

i-j	R_{ij}^{0}(Å)	w_{ij}
Zr-Hf	3.19	0.151
Hf-Hf	3.18	0.134
Hf-Cu	2.87	0.111
Hf-Ni	2.84	0.107
Ti-Hf	3.06	0.081
Zr-Cu	2.88	0.063
Zr-Ni	2.85	0.060
Ti-Zr	3.07	0.046
Cu-Ni	2.53	0.044
Zr-Zr	3.2	0.043
Ti-Cu	2.75	0.034
Ti-Ni	2.72	0.032
Cu-Cu	2.56	0.023
Ni-Ni	2.5	0.021
Hf-Be	2.72	0.014
Ti-Ti	2.94	0.012
Zr-Be	2.73	0.008
Cu-Be	2.41	0.006
Ni-Be	2.38	0.005
Ti-Be	2.6	0.004
Be-Be	2.26	0.000

7. Anelastic recovery

The normalized decay curves of anelastic recovery can be fitted by the KWW equation：

$$\frac{\varepsilon_{an}(t)-\varepsilon_{an}(\infty)}{\varepsilon_{an}(0)-\varepsilon_{an}(\infty)}=\exp\left(-\left(\frac{t}{\tau_{rec}}\right)^{\beta_{rec}}\right)$$

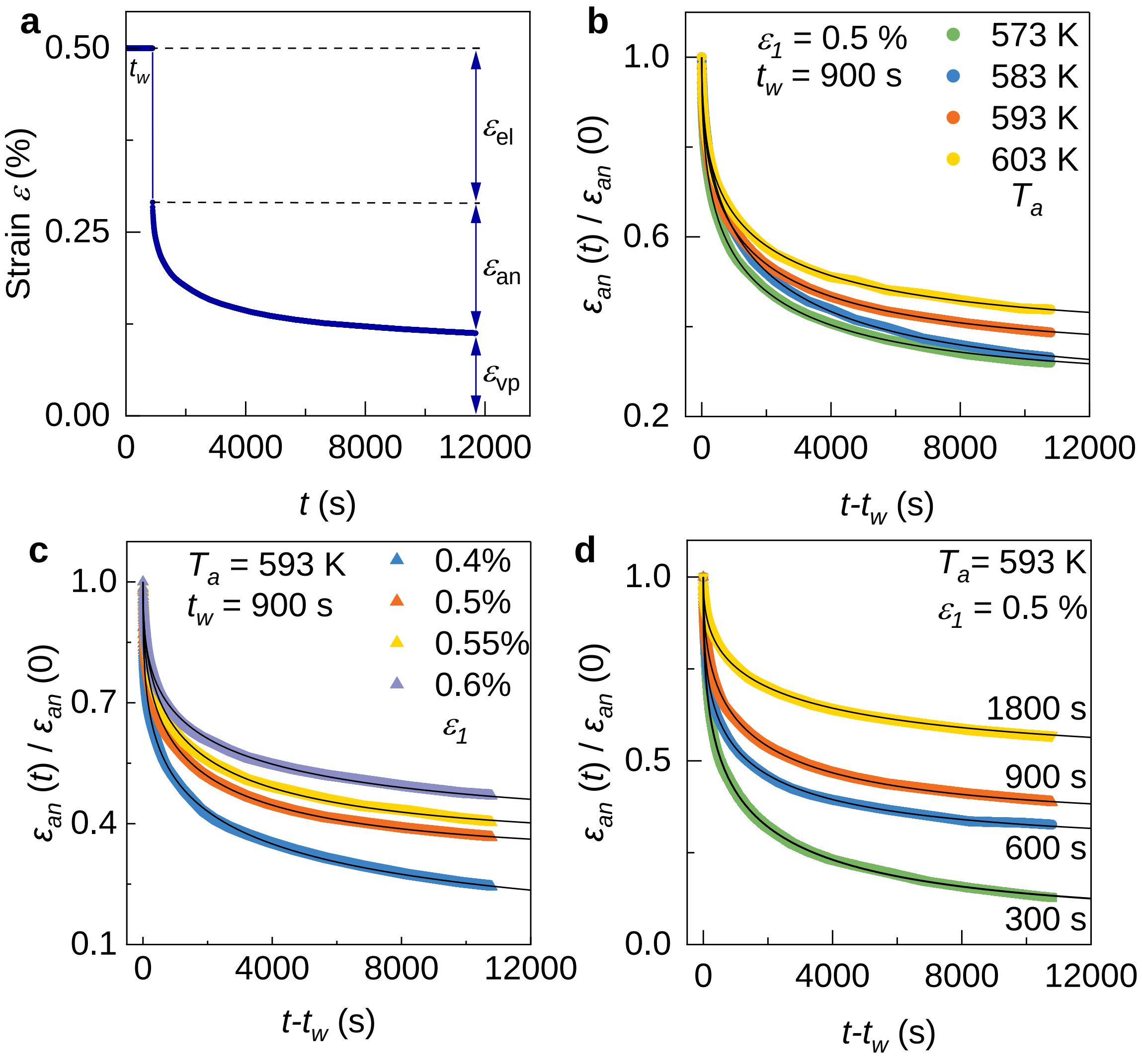

Supplementary Fig. 9: Solw relaxation of anelastic recovery of TiZrHfCuNiBe MG. a Schematic of the three components of the deformation observed in recovery experiment without applied force after stress relaxation below T_g (ε_{el}: elastic, ε_{an}: anelastic, ε_{vp}: viscoplastic); The effect of the various independent variables: **b** temperature T_a, **c** preloading strain ε_1, **d** preloading time t_w on normalized decay curves of anelastic recovery. The solid curves are KWW fitting. Source data are provided as a Source Data file.
